# HuR keeps an angiogenic switch on by stabilising mRNA of VEGF and COX-2 in tumour endothelium

**DOI:** 10.1038/bjc.2011.20

**Published:** 2011-02-01

**Authors:** T Kurosu, N Ohga, Y Hida, N Maishi, K Akiyama, W Kakuguchi, T Kuroshima, M Kondo, T Akino, Y Totsuka, M Shindoh, F Higashino, K Hida

**Affiliations:** 1 Department of Vascular Biology, Graduate School of Dental Medicine, N13 W7, Kita-ku, Sapporo 060-8586, Japan; 2 Department of Oral Pathology and Biology, Graduate School of Dental Medicine, Sapporo, Japan; 3 Department of Oral and Maxillofacial Surgery, Graduate School of Dental Medicine, Sapporo, Japan; 4 Department of Surgical Oncology, Graduate School of Medicine, Hokkaido University, Sapporo, Japan

**Keywords:** tumour endothelial cells, VEGF-A, COX-2, HuR, mRNA, angiogenesis

## Abstract

**Background:**

Tumour stromal cells differ from its normal counterpart. We have shown that tumour endothelial cells (TECs) isolated from tumour tissues are also abnormal. Furthermore, we found that mRNAs of vascular endothelial growth factor-A (VEGF-A) and cyclooxygenase-2 (COX-2) were upregulated in TECs. Vascular endothelial growth factor-A and COX-2 are angiogenic factors and their mRNAs contain an AU-rich element (ARE). AU-rich element-containing mRNAs are reportedly stabilised by Hu antigen R (HuR), which is exported to the cytoplasm.

**Methods:**

Normal endothelial cell (NEC) and two types of TECs were isolated. We evaluated the correlation of HuR and accumulation of VEGF-A and COX-2 mRNAs in TECs and effects of HuR on biological phenotypes of TECs.

**Results:**

The HuR protein was accumulated in the cytoplasm of TECs, but not in NECs. Vascular endothelial growth factor-A and COX-2 mRNA levels decreased due to HuR knockdown and it was shown that these ARE-mRNA were bound to HuR in TECs. Furthermore, HuR knockdown inhibited cell survival, random motility, tube formation, and Akt phosphorylation in TECs.

**Conclusion:**

Hu antigen R is associated with the upregulation of VEGF-A and COX-2 mRNA in TECs, and has an important role in keeping an angiogenic switch on, through activating angiogenic phenotype in tumour endothelium.

Tumour stroma includes various types of cells, such as leukocytes, lymphocytes, macrophages, fibroblasts, and endothelial cells (ECs). Tumour stromal cells are different from their normal counterparts. For example, cancer-associated fibroblasts promote cancer progression (Kalluri and Zeisberg, 2006) and tumour-associated macrophages produce epithelial growth factor, which stimulate tumour cell migration in breast cancer (Wyckoff *et al*, 2004). We previously found that tumour endothelial cells (TECs), which are also components of the tumour stroma, are different from normal endothelial cells (NECs) (Hida *et al*, 2004; Hida and Klagsbrun, 2005; Ohga *et al*, 2009). For example, TECs are cytogenetically abnormal (Hida *et al*, 2004; Akino *et al*, 2009), and they express specific markers such as tumour endothelial markers (TEMs) and upregulate growth factor receptor, such as vascular endothelial growth factor receptor (VEGFR) or epithelial growth factor receptor (Hida *et al*, 2008). Also, TECs show higher proliferation and resistance to serum starvation (Matsuda *et al*, 2010). Furthermore, they upregulate mRNA of VEGF-A and cyclooxygenase (COX)-2, which are generally expressed in tumour and other stromal cells. This suggests the presence of a functional autocrine pathway related to VEGF-A or COX-2 in mouse TECs.

Vascular endothelial growth factor is released by various normal and transformed cells under certain conditions such as hypoxia or growth factor stimulation, but particularly under the tumour microenvironment. Vascular endothelial growth factor-A is the most potent inducer of EC proliferation, migration, and survival (Ferrara and Davis-Smyth, 1997). Both tumour and stromal VEGF contribute to tumour angiogenesis.

Cyclooxygenase is a rate-limiting enzyme in the prostaglandin (PG) biosynthetic pathway. Cyclooxygenase consists of the following two isoforms: COX-1, which is expressed constitutively in most cells and COX-2, which is inducible by mitogens and proinflammatory cytokines during pathological processes, including inflammation (Hla *et al*, 1993). Cyclooxygenase-2 is also induced by growth factors, including VEGF (Hernandez *et al*, 2001; Tamura *et al*, 2002) and basic fibroblast growth factor (Kage *et al*, 1999). Thus, COX-2 is an important mediator of angiogenesis and tumour growth (Gately and Li, 2004).

Recent studies have shown that PGE_2_ (a downstream product of COX-2 metabolism) stimulates VEGF expression in ECs (Pai *et al*, 2001), and COX-2 induced by VEGF also has an important role in tumour angiogenesis (Wu *et al*, 2006). Thus, VEGF and COX-2-dependent PGE_2_ are important for angiogenesis (Tamura *et al*, 2006).

The mRNAs transcribed from VEGF-A and COX-2 genes include an AU-rich element (ARE). Several proto-oncogenes, such as c-myc or c-fos, possess an ARE in the 3′-untranslated regions of their mRNA. These mRNAs are stabilised by Hu antigen R (HuR) protein (Lopez de Silanes *et al*, 2005), a ubiquitously expressing protein belonging to the ELAV-like family of RNA-binding proteins, which regulates the expression of labile ARE-mRNAs by enhancing their stability and translation (Brennan and Steitz, 2001). Hu antigen R is predominantly localised in the nucleus of most unstimulated cells; however, it can also translocate to the cytoplasm with the target mRNAs and prevent their decay under stress such as heat shock (Gallouzi *et al*, 2001) and hypoxia (Levy *et al*, 1998). Although the precise transport mechanism remains unknown, it differs in tumour cells compared with that in normal cells (Higashino *et al*, 2005; Hasegawa *et al*, 2009). Cytoplasmic HuR expression has been implicated in the malignancy of several tumours (Lopez de Silanes *et al*, 2003, 2005; Denkert *et al*, 2004; Erkinheimo *et al*, 2005; Heinonen *et al*, 2005; Cho *et al*, 2007a, 2008b; Niesporek *et al*, 2008; Hasegawa *et al*, 2009). However, there are no reports about the function of HuR in vascular ECs.

In this study, we evaluated the correlation of HuR and accumulation of VEGF-A and COX-2 mRNAs in TECs. Furthermore, we analysed how HuR affects biological phenotypes of TECs.

## Materials and methods

### Cell lines and culture conditions

Cells from super-metastatic human malignant melanoma cell line A375SM, kindly donated by Dr IJ Fidler (MD Anderson Cancer Center, Houston, TX, USA) were cultured in a humidified atmosphere of 5% CO_2_ and 95% air at 37 °C in minimum essential medium (Gibco, Grand Island, NY, USA) supplemented with 10% heat-inactivated fetal bovine serum (FBS). The human oral carcinoma cell line HSC-3 was supplied by the Japanese Cancer Research Bank (Tokyo, Japan). The cells were cultured in Dulbecco's modified Eagle's medium (Sigma-Aldrich, St Louis, MO, USA) supplemented with 10% FBS. Human dermal microvascular endothelial cells (HMVECs) were purchased (Lonza, Walkersville, MD, USA) and cultured in endothelial growth medium (EGM-2 MV; Lonza) and 5% FBS.

### Antibodies

The antibodies purchased were rat anti-mouse CD31 and fluorescein isothiocyanate (FITC)-anti-mouse CD31 (eBioscience, San Diego, CA, USA), FITC-Bandeirea simplicifolia lectin 1-B4 (BS1-B4; Vector Laboratories, Burlingame, CA, USA), FITC-anti-rat IgG, anti-HuR (3A2) (Santa Cruz Biotechnology, Santa Cruz, CA, USA); PE-anti-human CD31 (eBioscience); FITC-anti-rabbit IgG (eBioscience); rabbit polyclonal to VEGF-A (Abcam, Cambridge, MA, USA); COX-2 (Cell Signaling, Beverly, MA, USA); anti-monoclonal*β*-actin-peroxidase (Sigma-Aldrich); anti-hnRNP A1(E-17):SC10030 (Santa Cruz) (Santa Cruz Biotechnology), anti-*β*-tubulin (Millipore, Bivellica, MA, USA) p-Akt (Cell Signaling Technology), Akt (Cell Signaling Technology); HRP-conjugated goat anti-rabbit IgG antibody (Cell Signaling), HRP-conjugated goat anti-mouse IgG antibody (Jackson ImmunoResearch), HRP-conjugated mouse anti-goat antibody (Jackson ImmunoResearch).

### Isolation of TECs and NECs

All animal procedures were performed in compliance with the guidelines prescribed by Hokkaido University, and protocols were approved by the Institutional Animal Care and Use Committee. Endothelial cells were isolated as described previously (Hida *et al*, 2004). Briefly, TECs were isolated from melanoma (A375SM) and oral carcinoma (HSC-3) xenografts of nude mice aged 8–12 weeks (Sankyo Labo Service Corporation, Inc., Tokyo, Japan). Normal endothelial cells were isolated from the dermal tissue and used as controls. Endothelial cells were isolated with a magnetic cell sorting system (Miltenyi Biotec, Bergisch Gladbach, Germany) according to the manufacturer's instructions, using the FITC-anti-CD31 antibody. CD31-positive cells were sorted and plated onto 1.5% gelatin-coated culture plates and grown in EGM-2 MV with 5% FBS. Diphtheria toxin (Calbiochem, San Diego, CA, USA) (500 ng ml^–1^) was added to TEC subcultures to kill any remaining human tumour cells (Arbiser *et al*, 1999), and to NECs to ensure technical consistency. The isolated ECs were purified by a second round of purification using FITC-BS1-B4, and purity was determined using flow cytometry (Hida *et al*, 2004). Tumour endothelial cells and NECs were characterised as described previously (Akino *et al*, 2009; Ohga *et al*, 2009; Matsuda *et al*, 2010). Tumour endothelial cells are positive for EC markers (VEGFR-1, VEGFR-2, CD31, CD144) and negative for haematopoietic markers (CD11b, CD45) (Supplementary Figure S1). All ECs were used between 15 and 20 passages.

### HuR knockdown

Hu antigen R siRNA was transfected using HiPerFect transfection reagent (Qiagen, Hilden, Germany) according to the manufacturer's instructions. The *HuR* mRNA and protein knockdown level was analysed using qRT–PCR and western blot analysis. The HuR siRNA was 5′-UUACCAGUUUCAAAUGGUCATT-3′ (Hasegawa *et al*, 2009; Kakuguchi *et al*, 2010), and the control siRNA was AllStars negative control siRNA (Qiagen).

### Cell fractionation

Cell fractionation was performed by separating cells into cytoplasmic and nuclear fractions, as described previously (Weigel and Dobbelstein, 2000). The cells were harvested and resuspended in a fractionating buffer (10 mM Tris–HCl, pH 7.6; 150 mM NaCl; 1.5 mM MgCl_2_, 0.5% Nonidet P-40 (Sigma-Aldrich), and protease inhibitor cocktail), followed by vigorous shaking for 5 min and centrifuged at 12 000 r.p.m. for 30 s. The supernatant was used as the cytoplasmic fraction. The accuracy of cell fractionation was confirmed by immunoblotting using cytoplasmic protein; *β*-tubulin and nuclear protein; hnRNP.

### Quantitative real-time RT–PCR

Total RNA was isolated from ECs using the RNeasy Micro kit (Qiagen) using the RNase-free DNase Set (Qiagen). RNA was quantified using spectrophotometry. Total RNA was then used for performing first-strand complementary DNA synthesis by using the ReverTra-Plus (Toyobo, Osaka, Japan). Real-time PCR was conducted using the SYBR Green Realtime PCR Master Mix Plus (Toyobo). Cycling conditions were set according to the manufacturer's instructions based on the use of Opticon Monitor version 3.0 (Bio-Rad, Hercules, CA, USA).

To evaluate the half-life of VEGF-A and COX-2 mRNA, oral carcinoma EC and melanoma EC and skin EC were treated with actinomycin D (Act. D) (Sigma-Aldrich) (5 *μ*g ml^–1^) for the indicated time periods. The extracted RNA was subjected to quantitative real-time RT–PCR. The VEGF-A and COX-2 mRNA expression levels in ECs were normalised to GAPDH levels. The experiment was performed three times and similar results were obtained. The primers used were as follows: GAPDH, forward 5′-TCTGACGTGCCGCCTGGAG-3′, reverse 5′-TCGCAGGAGACAACCTGGTC-3′ VEGF-A, forward 5′- GATTGAGACCCTGGTGGACATC-3′, reverse 5′- CACACAGGAGGGCTTGAAGA-3′ *COX-2*, forward 5′- CAGACAACATAAACTGCGCCTTTT-3′, reverse 5′- GACTTCCTGCCCCACAGCAA-3′ HuR, forward 5′-CCTCCGAGCCCATCACAGT-3′, reverse 5′-GCGAGAGGAGAGCCATGTTT-3′.

### RIP assay

Ribonucleoprotein (RNP) immunoprecipitation assay was performed as described previously (Higashino *et al*, 2005). Tumour endothelial cells were treated with PBS containing 1% formaldehyde and the lysate was immunoprecipitated with mouse IgG (eBioscience) or anti-HuR antibody (Santa Cruz). The pellets were incubated at 70 °C for 45 min to reverse the cross-links, the isolated RNA was subjected to reverse transcription, and PCR amplification for VEGF-A, COX-2 was performed using the primers as described above.

### Immunocytochemistry and immunohistochemistry

Human tissue samples were obtained from excised renal cell carcinoma and normal renal tissue of four patients at Hokkaido University Hospital, Hokkaido, Japan, and from normal renal tissues. Informed consent was obtained from all patients before the samples were used.

Frozen sections were fixed in cold acetone for 15 min and blocked with 2% goat and 5% sheep serum in PBS for 1 h at room temperature. Serial sections were incubated with primary antibodies (CD31, HuR) for 16 h.

Immunohistochemical detections of CD31 and HuR in serial sections were carried out using the avidin–biotin complex method as previously described (Shindoh *et al*, 1996).

Furthermore, the cryosections of renal carcinoma and normal renal tissue were double stained using PE-anti-human CD31 antibody and anti-human HuR antibody, Alexa488-conjugated anti-mouse IgG antibody to show co-localisation of HuR in EC. Nuclei were stained with 4′,6-diamidino-2-phenylindole (DAPI). In addition, cultured ECs were fixed in methanol and stained with anti-HuR antibody and then with FITC-conjugated secondary antibody. All samples in immunocytochemistry were counterstained with DAPI (Roche, Indianapolis, IN, USA). The cells were observed using an OLYMPUS IX71 fluorescence microscope (Olympus, Tokyo, Japan).

### Western blot analysis

Western blot analysis was performed using antibodies specific to HuR, VEGF-A, COX-2, p-Akt, Akt, *β*-actin, and HRP-conjugated secondary antibody as described previously (Hasegawa *et al*, 2009; Ohga *et al*, 2009). The accuracy of cell fractionation was confirmed by immunoblotting using cytoplasmic protein; *β*-tubulin and nuclear protein; hnRNP (Hasegawa *et al*, 2009). The levels of VEGF-A and COX-2 were normalised to *β*-actin and analysed by scanning densitometry using Image J software from the NIH (Bethesda, MD, USA).

### Cell proliferation (survival) assay

Tumour endothelial cells were treated with control siRNA (5 nM), HuR siRNA (5 nM), and without siRNA. After siRNA transfection for 24 h, 5 × 10^4^ cells per well were seeded into 12-well dishes in EBM-2 with 0.5% FBS (low serum medium), and then VEGF (10 ng ml^–1^) or PGE_2_ (10 nM) was added. Tumour endothelial cells were trypsinised and the cell number was counted 48 h after cell seeding. The experiment was performed three times and similar results were obtained. Statistical analysis was performed using the Mann–Whitney *U*-test. A *P*-value of <0.05 was considered significant.

### Cell migration assay

Random motility of TECs was measured by a migration assay using a Boyden chamber as described previously (Dormond *et al*, 2001). Tumour endothelial cells were treated with control siRNA (5 nM), HuR siRNA (5 nM), or without siRNA in low serum medium for 48 h. In all, 1.5 × 10^4^ cells were seeded in the upper chambers in low serum medium, and then VEGF (10 ng ml^–1^) or PGE_2_ (10 nM) was added. No chemoattractant was added to the lower chambers.

### Tube formation assay

Diluted Matrigel (BD Biosciences, San Jose, CA, USA) was transferred to each well of a 24-well dish and incubated at 37 °C for 30 min to allow the matrix solution to solidify. Tumour endothelial cells were harvested and resuspended in appropriate media and then seeded at a density of 1 × 10^5^ cells per well, followed by incubation at 37 °C for 12 h. Tube formation was observed using an inverted microscope and the experimental results were recorded at different times. The number of tube junctions/areas was counted.

## Results

### VEGF-A and COX-2 were upregulated in TECs not in NECs

**Figure 1:**
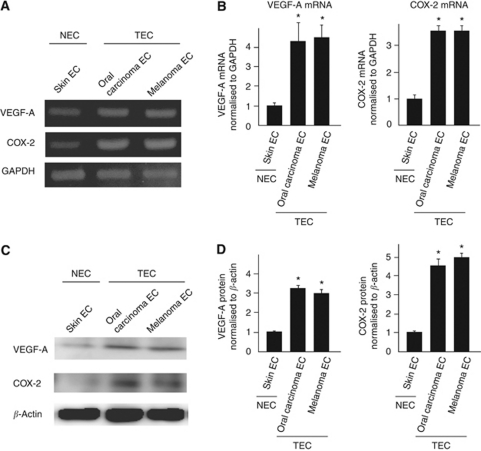
VEGF-A and COX-2 were upregulated in TECs. COX-2 and VEGF-A mRNA levels in regular RT–PCR (**A**). Relative expression of VEGF-A and COX-2 mRNAs in TECs (oral carcinoma and melanoma ECs) and NEC (skin EC) was measured using quantitative real-time RT–PCR (**B**). VEGF-A and COX-2 mRNA levels were significantly higher in TECs than in NECs (^*^P<0.05 *vs* skin EC). Western blotting of VEGF-A and COX-2 also showed that VEGF-A and COX-2 proteins expression were upregulated in TECs (**C**). The levels of VEGF-A and COX-2 were normalised to *β*-actin and analysed by scanning densitometry using Image J software from the NIH (^*^P<0.01 *vs* skin EC) (**D**).

Vascular endothelial growth factor-A and COX-2 are upregulated in tumour cells; however, only a few studies report about their expression in TECs (Masferrer *et al*, 2000; Leahy *et al*, 2002). We previously isolated TECs from four kinds of tumour xenografts in nude mice, and observed that TECs as well as NECs expressed typical EC markers such as CD31, CD105, and CD144. Using these isolated ECs, we reported that TECs are different from NECs (Hida *et al*, 2004, 2008; Hida and Klagsbrun, 2005). For example, TECs are more proliferative and migratory than NECs. In addition, gene expression pattern in TECs is different from that in NECs. Our recent microarray analysis showed that several genes in TECs, such as VEGFR-2 and reported TEC markers, including TEM-8 (St Croix *et al*, 2000), CD13 (Pasqualini *et al*, 2000), and Dkk-3 (Untergasser *et al*, 2008; Fong *et al*, 2009), were expressed excessively. Of these, we have chosen VEGF-A and COX-2 mRNAs to examine their expression in TECs. The levels of VEGF-A and COX-2 mRNAs in TECs were several folds higher than those in NECs, based on the results from regular RT–PCR (Figure 1A) and real-time PCR (Figure 1B) analyses. We also found that VEGFR-1 or -2 mRNAs were upregulated in TECs (data not shown). In addition, western blotting of VEGF-A and COX-2 also showed that VEGF-A and COX-2 proteins expression were upregulated in TECs (Figures 1C and D).

These findings suggested that VEGF-A and COX-2 expression have an important role in the increased proliferative rate of TECs.

### Differential localisation of HuR between TECs and NECs *in vitro* and *in vivo*

Since VEGF-A and COX-2 mRNAs are ARE-mRNAs, we next focused on HuR. Hu antigen R is only localised in the nucleus of normal cells, but it is localised also in the cytoplasm of cells under stress – such as heat shock or hypoxia – or in the cytoplasm of malignant cells (Levy *et al*, 1998; Gallouzi *et al*, 2001).

**Figure 2:**
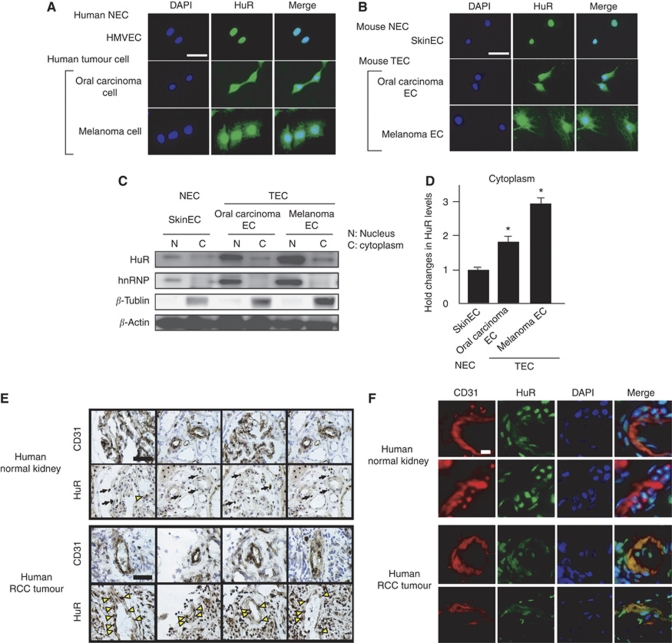
Differential localisation of the HuR protein between TECs and NECs. HuR localisation was analysed by fluorescent immunocytochemistry. HuR was localised only in the nucleus of HMVEC, whereas it was expressed in the cytoplasm as well of oral carcinoma and melanoma cells (**A**). HuR expression was limited to the nuclei of mouse normal ECs (skin EC); however, it was detected not only in the nucleus but also in the cytoplasm of mouse tumour ECs (oral carcinoma and melanoma ECs) (**B**). Blue: DAPI, Green: HuR (white bar: 50 *μ*m). To confirm the cytoplasmic localisation of HuR in TEC, ECs were separated into cytoplasmic and nuclear fractions. Using two antibodies, anti-hnRNP (nuclear protein) and anti-*β*-tubulin (cytoplasmic protein), it was confirmed that nuclear and cytoplasmic protein extracts were pure in each fraction. The HuR of each fraction was detected by western blotting. The amounts of HuR in the cytoplasm of TECs were much higher than NECs (^*^P<0.05 *vs* skin EC) (**C** and **D**). HuR expression was analysed in human tissue sections. HuR was expressed only in nuclei in human NECs (white arrow); however, it was expressed in cytoplasm in human TECs (yellow arrow), which were stained by anti-CD31 antibody (**E**). (Black bar: 50 *μ*m) Immunofluorescent double staining with anti-CD31 and anti-HuR antibodies in the frozen sections of human renal tumours and normal renal tissues. HuR staining is stained in cytoplasm in TECs stained with anti-CD31, but only in nuclei in NEC (**F**). (White bar: 10 *μ*l).

Consistent with the results in the previous reports about HuR localisation in normal and malignant cells, HuR was expressed only in the nucleus of HMVECs; however, it was also expressed in the cytoplasm and nucleus of malignant melanoma and oral carcinoma cells (Figure 2A). To examine the HuR localisation in TECs and NECs, we isolated mouse ECs (Figure 2B). Hu antigen R expression was detected only in the nucleus of NECs with anti-HuR antibody, similar to that in HMVECs. In contrast, both the cytoplasm and nucleus of TEC, which were isolated from oral carcinoma and melanoma ECs, were stained positively with anti-HuR antibody (Santa Cruz Biotechnology).

To confirm the cytoplasmic localisation of HuR in TEC, ECs were separated into the cytoplasmic and nuclear fractions. Using two antibodies, anti-hnRNP (nuclear protein) and anti-*β*-tubulin (cytoplasmic protein), we could confirm that nuclear and cytoplasmic protein extracts were pure in each fraction.

The HuR of each fraction was detected by western blotting. The amounts of HuR in the cytoplasm of TECs were much higher than that in NECs (Figures 2C and D). These results also suggest that HuR is accumulated in the TEC cytoplasm, similar to the malignant melanoma cells.

We next examined HuR localisation in a human tumour or normal blood vessels by immunohistochemistry using serial frozen sections of human tumours and normal kidney tissues. In normal blood vessels, which were stained by anti-CD31 (BD Biosciences), HuR expression was localised only in the nuclei of ECs (black arrow, Figure 2E). On the other hand, HuR expression was detected not only in the nuclei and cytoplasm of TECs, which were stained with CD31 (yellow arrow, Figure 2E). Furthermore, in immunofluorescent double staining with anti-CD31 and anti-HuR antibodies in same frozen sections of human renal tumours and normal renal tissues, HuR is stained in cytoplasm in TECs stained with anti-CD31, but only in nuclei in NEC, consistently with Figures 2E and F. These results suggested that HuR was expressed in the cytoplasm of both mouse TECs and human TECs, similar to malignant cells.

### HuR knockdown reduces VEGF-A and COX-2 expression in TECs

**Figure 3:**
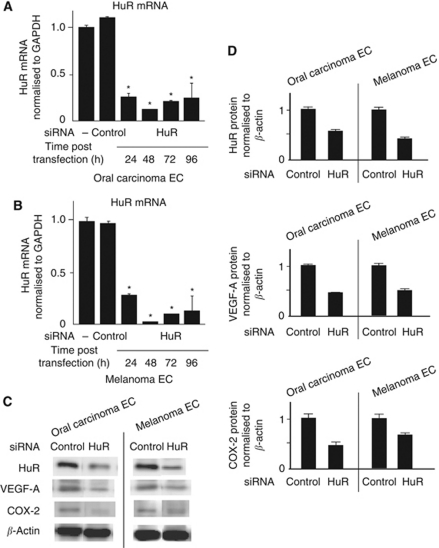
HuR knockdown by siRNA in TECs. Oral carcinoma and melanoma ECs were transfected with siRNA to downregulate HuR expression. Knockdown of HuR mRNA was confirmed 24–96 h after transfection using quantitative real-time RT–PCR (**A** and **B**) (^*^P<0.05 *vs* control si). Silence of HuR protein was determined by western blot and the expression of VEGF-A and COX-2 protein was also downregulated in HuR-knockdown TECs (**C** and **D**) (^*^P<0.05 *vs* skin EC).

To explore the role of HuR in the expression of VEGF-A and COX-2 mRNA of TECs, TECs were subjected to RNAi using HuR siRNA to silence HuR expression (Hasegawa *et al*, 2009; Kakuguchi *et al*, 2010). Hu antigen R knockdown both in oral carcinoma and melanoma ECs was confirmed in its mRNA level (Figures 3A and B) and in its protein level. The expression of VEGF-A and COX-2 protein were also downregulated in HuR-knockdown TECs (Figures 3C and D).

**Figure 4:**
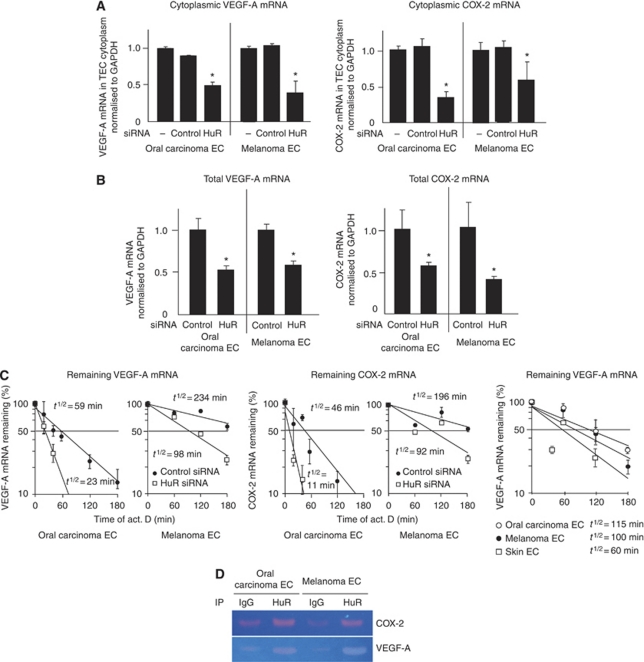
Cytoplasmic and total VEGF-A and COX-2 mRNA levels were decreased in TECs by HuR knockdown. Cytoplasmic mRNAs were isolated from TECs transfected with siRNA 48 h after the transfection. Relative expression of VEGF-A and COX-2 mRNAs in the cytoplasm of oral carcinoma and melanoma ECs was measured using quantitative real-time RT–PCR. The graph shows the relative rate of cytoplasmic mRNA/total mRNA. Cytoplasmic VEGF-A and COX-2 mRNA levels decreased in TECs by HuR knockdown (**A**) (^*^P<0.05 *vs* control si). VEGF-A and COX-2 mRNA in the total cell (nuclei+cytoplasm) were also downregulated by HuR knockdown (**B**) (^*^P<0.05 *vs* control si). The cells were treated with Act. D, and the amount of each ARE-mRNA was estimated at the indicated time by quantitative real-time RT–PCR. The half-life of these mRNAs was shortened by HuR knockdown. In addition, it was shown that the half-life of VEGF-A mRNA was longer in TEC compared with NEC (**C**). VEGF-A and COX-2 mRNA associated with HuR was isolated by RNP immunoprecipitation analysis. Both mRNAs were immunoprecipitated by anti-HuR antibody (**D**).

The quantity of VEGF-A and COX-2 mRNA in the cytoplasm of HuR-knockdown cells was reduced to almost half of the amount of TECs in which control siRNA (Qiagen GmbH) was introduced (Figure 4A), suggesting that HuR contributes to the export of VEGF-A and COX-2 mRNAs in TECs. The accumulation of VEGF-A and COX-2 mRNA in both cells was also downregulated in HuR-knockdown cells (Figure 4B). Furthermore, the half-lives of VEGF-A and COX-2 mRNA were shortened by HuR knockdown (Figure 4C). It was expected that these mRNA stability was greater in TEC compared with NEC, since cytoplasmic HuR expression of TEC was higher than that of NEC. It was shown that the half-life of VEGF-A mRNA was longer in TEC compared with NEC as expected (Figure 4C). To address whether HuR indeed associates VEGF-A or COX-2 mRNA directly, we performed a RIP assay. Since the bands of VEGF-A and COX-2 mRNAs were visible in the samples co-precipitated with HuR (Figure 4D), the data indicate that HuR binds to these mRNAs. These results suggest that HuR contributes to the stabilisation of VEGF-A and COX-2 mRNAs by binding to these mRNAs.

### TEC survival was inhibited by HuR knockdown

Vascular endothelial growth factor-A and COX-2 are proangiogenic factors and affect biological cellular phenotypes such as proliferation, migration, and survival (Ferrara and Davis-Smyth, 1997; Williams *et al*, 1999).

**Figure 5:**
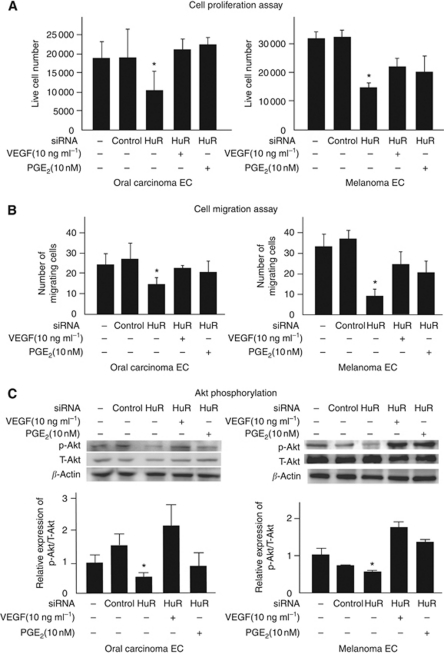
TEC survival, cell motility, and Akt phosphorylation were inhibited by HuR knockdown. TEC survival was analysed under low serum condition by counting the number of live cells at 72 h after siRNA transfection (**A**). TEC survival rate under serum-starved conditions decreased significantly by HuR knockdown (^*^P<0.05 *vs* skin EC). Random motility of TECs was analysed using Boyden chamber migration assay (**B**). After siRNA transfection for 48 h, endothelial basal media without growth factors (0.5% serum) was added to both the upper and lower wells to analyse random cell motility, and migrating cells moving towards the lower well were counted 6 h later. Cell motility was inhibited significantly by HuR knockdown (^*^P<0.05 *vs* skin EC). Cell motility was restored when TECs were treated with VEGF (10 ng ml^–1^) or PGE_2_ (10 nM). The levels of phosphorylated Akt (P-Akt) were determined by western blotting using an anti-P-Akt antibody (**C**, top lane). The membrane was stripped and reincubated with anti-total Akt (T-Akt) antibody (**C**, middle lane) and *β*-actin antibody (**C**, bottom lane) to detect the quantity of T-Akt and *β*-actin protein. Levels of P-Akt were normalised to T-Akt using densitometry. The graph shows the relative ratio of P-Akt/T-Akt analysed using densitometry. HuR knockdown inhibited Akt phosphorylation.

To analyse the effects of VEGF-A and COX-2 downregulation mediated by HuR knockdown in TECs, we performed a cell proliferation assay under a low serum condition. Hu antigen R knockdown produced starved oral carcinoma and melanoma ECs compared with TECs, which were untreated or transfected with control siRNA (Figure 5A). Interestingly, when treated with VEGF (10 ng ml^–1^; Lonza) or PGE_2_ (10 nM; Cayman Chemical, Ann Arbor, MI, USA), cell proliferation of both TECs, even those transfected with siHuR, were restored. These results suggest that VEGF and PGE_2_-treated TECs survive under serum starvation, and HuR contributes to this survival, at least in part, by upregulating VEGF-A and COX-2 mRNAs.

### HuR knockdown inhibits random motility and Akt phosphorylation in TECs

To analyse another effect of HuR knockdown on angiogenic properties of TECs, we performed a cell migration assay using a Boyden chamber (Neuro Probe Inc., Gaithersburg, MD, USA) (Figure 5B). No chemoattractant was added to the upper and lower chambers, which enabled us to assess the random motility of TECs. The migrating cell number decreased significantly by HuR knockdown both in oral carcinoma and melanoma ECs. When VEGF (10 ng ml^–1^) or PGE_2_ (10 nM) was added to the upper chamber, the number of cells that migrated towards the lower chamber was restored.

During angiogenesis, VEGF signalling is mediated by ligand-dependent signalling through the PI3K/Akt pathway (Rodriguez *et al*, 2006). The protein kinase Akt has a central role in mature ECs. Activation of Akt promotes cell survival by inhibiting apoptosis (Gerber *et al*, 1998) and mediates VEGF-induced migration (Dimmeler *et al*, 2000; Morales-Ruiz *et al*, 2000). Cyclooxygenase-2-overexpressed cells produce PGs that stimulate both EC migration and tube formation (Tsujii *et al*, 1998). Hence, we analysed the effect of HuR knockdown on Akt phosphorylation in TECs (Figure 5C). Hu antigen R knockdown inhibited Akt phosphorylation in both TECs, whereas treatment with VEGF and PGE_2_ for 30 min recovered Akt phosphorylation. These results suggested that VEGF-A and COX-2 mRNAs, which are exported to the cytoplasm by HuR, have important roles in TEC motility through Akt signalling.

### HuR knockdown inhibits TEC tube formation

**Figure 6:**
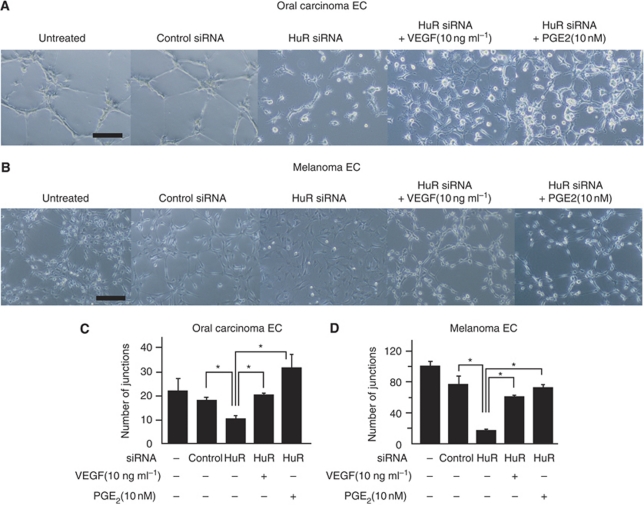
Tube formation of TECs was inhibited by HuR knockdown. TECs were seeded onto Matrigel in basal medium, and tube formation was observed after 12 h incubation (**A** and **B**) and the number of junctions counted (**C** and **D**) (^*^P<0.01 *vs* HuR SiRNA. Tube formation was significantly inhibited by HuR siRNA both in oral carcinoma and melanoma ECs. When cells were treated with VEGF (10 ng ml^–1^) or PGE_2_ (10 nM), the number of junctions was restored. Representative figures are shown (black bar: 100 *μ*m).

The involvement of HuR in TEC angiogenic properties was investigated using a tube formation assay (Figure 6). Representative data are shown (Figure 6A and B). A quantitative analysis of junction numbers in tubes is also shown (Figure 6C). The ability to form capillary-like structures was impaired by HuR knockdown in oral carcinoma and melanoma ECs.

The ability to form capillaries in TECs was restored partially in the presence of VEGF or PGE_2_, suggesting that HuR is important for TEC tube formation. Hu antigen R knockdown suppresses angiogenic phenotypes of TECs and may cause an anti-angiogenic effect.

## Discussion

This study provided several results including the following: (1) VEGF-A and COX-2 mRNA were upregulated in mouse TECs isolated from tumour xenografts; (2) HuR was highly expressed in the cytoplasm of cultured mouse TECs and human TECs *in vivo*; (3) HuR bound to VEGF-A and COX-2 mRNAs and stabilised them in the TEC cytoplasm; (4) HuR knockdown led to the inhibition of cell survival, random motility, and tube formation in TECs; and (5) HuR knockdown suppressed Akt phosphorylation and TECs tube formation.

There are several reports about the relationship between HuR and ARE-mRNA (Brennan and Steitz, 2001) or the correlation between cytoplasmic HuR expression and malignancy in tumour cells (Lopez de Silanes *et al*, 2003, 2005; Denkert *et al*, 2004; Erkinheimo *et al*, 2005; Heinonen *et al*, 2005; Cho *et al*, 2007a, 2007b; Niesporek *et al*, 2008; Hasegawa *et al*, 2009). However, there are few reports about HuR and ARE-mRNA in ECs (Tschernatsch *et al*, 2006; Annabi *et al*, 2009), and no reports on the mechanism of accumulated VEGF-A or COX-2 mRNA expression in TECs. We have previously reported abnormalities of TECs (Hida *et al*, 2004; Hida and Klagsbrun, 2005; Ohga *et al*, 2009); they grow faster and migrate better than NECs (Matsuda *et al*, 2010). In our isolated mouse TECs, several genes, such as VEGFR-2, CD13 (Pasqualini *et al*, 2000), and Dkk-3 (Untergasser *et al*, 2008; Fong *et al*, 2009), which are reported to be the upregulated genes in TECs, were indeed upregulated. Furthermore, TECs are cytogenetically abnormal (Hida *et al*, 2004; Akino *et al*, 2009). They have a lower serum requirement, and although more responsive to angiogenic factors, they are more resistant to anti-cancer drug treatment, such as 5-fluorouracil (Hida *et al*, 2008). In this study, two angiogenic growth factors, VEGF-A and COX-2, were highly expressed in TECs compared with those in NECs, supporting previous findings about increased survival activity of TECs.

Since VEGF-A and COX-2 are ARE-mRNAs, we focused on the role of HuR in TECs. Several ARE-mRNAs, which are transcripts of oncogenes or growth factor genes, are upregulated in malignant cells. One of the accumulation mechanisms of these mRNAs is their stabilisation by HuR (Brennan and Steitz, 2001). In this study, we showed that HuR existed not only in the nucleus but also in the cytoplasm of TECs, and this result suggests that HuR was exported to the cytoplasm as reported in tumour cells. Furthermore, we showed that HuR knockdown caused decreased VEGF-A and COX-2 mRNA levels and shortened the half-life of these mRNAs, and their protein levels. In addition, we demonstrated that HuR binds to VEGF-A and COX-2 mRNAs by RIP assay. These results suggest that HuR contributes to the stabilisation of VEGF-A and COX-2 mRNAs in TEC cytoplasm.

In our data of western blotting, we used *β*-actin as an internal control. It was shown that *β*-actin expression level was changed by HuR knockdown in Hela cells (Dormoy-Raclet *et al*, 2007). However, there are also several reports showing that the expression of *β*-actin was not changed even when HuR level was changed in other types of tumour cells (Abdelmohsen *et al*, 2008; Ghosh *et al*, 2009; Hasegawa *et al*, 2009). Since *β*-actin was not changed in our case of TECs either, we used it as an internal control for western blotting, It was considered that the interaction between *β*-actin and HuR may be different depending on the cell type.

Hu antigen R knockdown inhibited survival, random motility, and tube formation in TECs and also inhibited Akt phosphorylation, suggesting that it has key roles in the TEC angiogenic phenotype by upregulating mRNAs of the angiogenic factors VEGF-A and COX-2. Since HuR reportedly stabilises not only VEGF-A and COX-2 mRNAs but also other ARE-mRNAs, it is possible that downregulation of survival, random motility, and tube formation in TECs occurred due to downregulation of other ARE-mRNAs. However, adding VEGF and PGE_2_ restored the angiogenic properties of TECs such as cell survival, migration, and tube formation with recovery of Akt phosphorylation, even with HuR knockdown. Hu antigen R-mediated VEGF-A and COX-2 mRNA upregulation has an important role in tumour angiogenesis. The VEGFR-1 and VEGFR-2 mRNA expression levels and Akt phosphorylation levels were higher in TECs than in NECs (Ohga *et al*, 2009). This suggests that there might be an autocrine pathway for cell survival and migration in TECs due to HuR translocation into the TEC cytoplasm. This result was not found in NECs.

Interestingly, human renal TECs were reported more proliferative and resistant to serum starvation with overexpressed VEGF-D and activated Akt phosphorylation, suggesting that TECs are stimulated in an autocrine manner (Bussolati *et al*, 2003).

In conclusion, we demonstrated for the first time that VEGF-A and COX-2 mRNAs are stabilised in TECs. Hu antigen R knockdown changed the features of TECs required for tumour angiogenesis. These results suggest that HuR may contribute to ARE-mRNA accumulation specifically in TECs and may keep an angiogenic switch on in TECs themselves. Hu antigen R knockdown may have potential as an effective therapeutic approach.

## Supplementary Material

Supplementary Figure s1
