# Supplementary figures and images for: HuR keeps an angiogenic switch on by stabilising mRNA of VEGF and COX-2 in tumour endothelium
Source: Br J Cancer. 2011 Feb 1;104(5):819–29. doi: 10.1038/bjc.2011.20 (PMC3048211; doi:10.1038/bjc.2011.20)

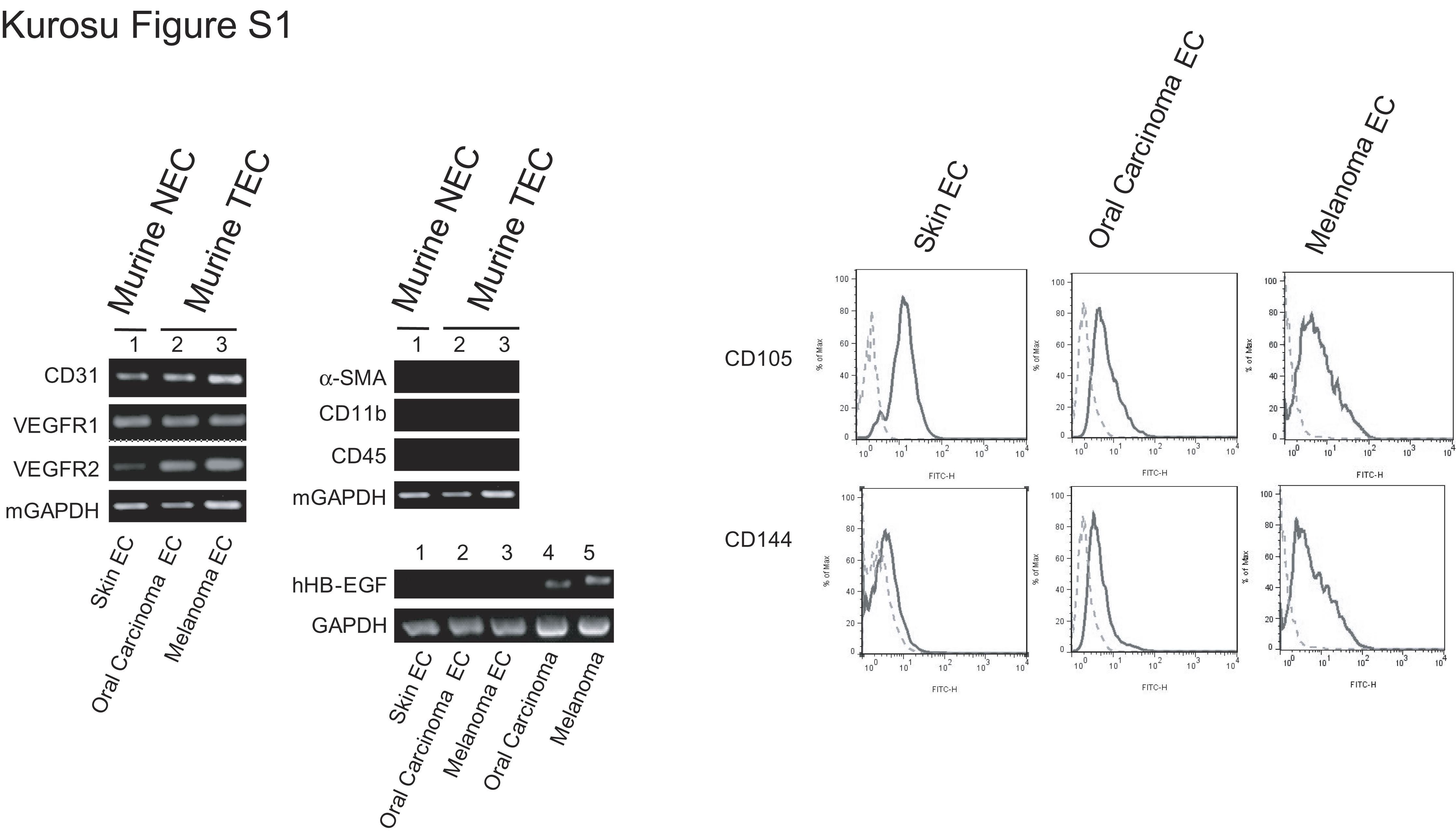

Supplement: Supplementary Figure s1 [file bjc201120x1.tif]
