# Supplementary material for: HuR keeps an angiogenic switch on by stabilising mRNA of VEGF and COX-2 in tumour endothelium
Source: Br J Cancer. 2011 Feb 1;104(5):819–29. doi: 10.1038/bjc.2011.20 (PMC3048211; doi:10.1038/bjc.2011.20)
Supplement: Supplementary Figure Legend [file bjc201120x2.doc]

Supplemental data

**Figure S1**

TECs and NECs were characterized by flow cytometry and RT-PCR. TEC are positive for EC markers；VEGFR1, VEGFR2, CD31, CD105, CD144. They were negative for hematopoietic markers; CD11b, CD45 and pericyte marker; α-SMA). Human HB-EGF (expressed in human tumor cells) was negative in TECs.
